# Association of MRI Volume Parameters in Predicting Patient Outcome at Time of Initial Diagnosis of Glioblastoma

**DOI:** 10.3390/brainsci13111579

**Published:** 2023-11-10

**Authors:** Kin Sing Lau, Isidoro Ruisi, Michael Back

**Affiliations:** 1 Department of Radiation Oncology, Northern Sydney Cancer Centre, Royal North Shore Hospital, Sydney, NSW 2065, Australia; kinsing.lau@health.nsw.gov.au; 2 Central Coast Cancer Centre, Gosford Hospital, Gosford, NSW 2250, Australia; 3 Genesis Care, Sydney, NSW 2015, Australia; 4 Sydney Medical School, University of Sydney, Sydney, NSW 2050, Australia; 5 The Brain Cancer Group, Sydney, NSW 2065, Australia

**Keywords:** glioblastoma, MRI volume, outcome

## Abstract

Purpose: Patients with glioblastoma (GBM) may demonstrate varying patterns of infiltration and relapse. Improving the ability to predict these patterns may influence the management strategies at the time of initial diagnosis. This study aims to examine the impact of the ratio (T2/T1) of the non-enhancing volume in T2-weighted images (T2) to the enhancing volume in MRI T1-weighted gadolinium-enhanced images (T1gad) on patient outcome. Methods and Materials: A retrospective audit was performed from established prospective databases in patients managed consecutively with radiation therapy (RT) for GBM between 2016 and 2019. Patient, tumour and treatment-related factors were assessed in relation to outcome. Volumetric data from the initial diagnostic MRI were obtained via the manual segmentation of the T1gd and T2 abnormalities. A T2/T1 ratio was calculated from these volumes. The initial relapse site was assessed on MRI in relation to the site of the original T1gad volume and surgical cavity. The major endpoints were median relapse-free survival (RFS) from the date of diagnosis and site of initial relapse (defined as either local at the initial surgical site or any distance more than 20 mm from initial T1gad abnormality). The analysis was performed for association between known prognostic factors as well as the radiological factors using log-rank tests for subgroup comparisons, with correction for multiple comparisons. Results: One hundred and seventy-seven patients with GBM were managed consecutively with RT between 2016 and 2019 and were eligible for the analysis. The median age was 62 years. Seventy-four percent were managed under a 60Gy (Stupp) protocol, whilst 26% were on a 40Gy (Elderly) protocol. Major neuroanatomical subsites were Lateral Temporal (18%), Anterior Temporal (13%) and Medial Frontal (10%). Median volumes on T1gd and T2 were 20 cm^3^ (q1–3:8–43) and 37 cm^3^ (q1–3: 17–70), respectively. The median T2/T1 ratio was 2.1. For the whole cohort, the median OS was 16.0 months (95%CI:14.1–18.0). One hundred and forty-eight patients have relapsed with a median RFS of 11.4 months (95%CI:10.4–12.5). A component of distant relapse was evident in 43.9% of relapses, with 23.6% isolated relapse. Better ECOG performance Status (*p* = 0.007), greater extent of resection (*p* = 0.020), MGMT methylation (*p* < 0.001) and RT60Gy Dose (*p* = 0.050) were associated with improved RFS. Although the continuous variable of initial T1gd volume (*p* = 0.39) and T2 volume (*p* = 0.23) were not associated with RFS, the lowest T2/T1 quartile (reflecting a relatively lower T2 volume compared to T1gd volume) was significantly associated with improved RFS (*p* = 0.016) compared with the highest quartile. The lowest T2/T1 ratio quartile was also associated with a lower risk of distant relapse (*p* = 0.031). Conclusion: In patients diagnosed with GBM, the volumetric parameters of the diagnostic MRI with a ratio of T2 and T1gad abnormality may assist in the prediction of relapse-free survival and patterns of relapse. A further understanding of these relationships has the potential to impact the design of future radiation therapy target volume delineation protocols.

## 1. Introduction

Predicting patterns of relapse of glioblastoma following management with adjuvant radiation therapy and temozolomide may potentially provide an opportunity to modify the initial management approach [1]. Molecular features such as MGMT methylation status have been demonstrated to influence this relapse pattern, with increased distant relapses occurring later in patients with an MGMT-methylated tumour [2,3]. However, despite different natural histories of tumours with different radiological appearances and varying patterns of relapse, the initial radiation therapy target volume delineation is identical in management guidelines [4,5]. 

The initial radiological appearance on diagnostic MRI may provide some information as to subsequent clinical behaviour and pattern of relapse. This study aims to determine the impact of the initial MRI sequences in patients diagnosed with IDH wildtype glioblastoma, specifically, the T1 gadolinium-enhanced volume and the associated T2 abnormality, on the subsequent survival outcomes and pattern of failure following adjuvant radiation therapy and temozolomide chemotherapy.

## 2. Methods

Newly diagnosed adult patients with a primary brain tumour referred to the Neuro-oncology Multidisciplinary Tumour Board at the Northern Sydney Cancer Centre from January 2016 to December 2019 were entered into a prospective database, approved by Institutional Ethics Review Board. Consecutive patients with glioblastoma (World Health Organization 2016 Grade IV glioma) managed with definitive or adjuvant chemoradiation consistent with the EORTC-NCIC (Stupp) protocol [5] or NCIC GBM Elderly protocol [6] were formally included for this study. All patients were managed as part of a multidisciplinary neuro-oncology tumour (MDT) team. Patient, tumour and treatment factors were recorded in a prospective database. 

### 2.1. Neuro-Oncological Management 

Patients were managed from multiple neurosurgical units, with an emphasis on optimising extent of resection through utilising techniques including endoscopic surgery and awake craniotomy. MRI scans were performed pre-operatively and generally in the 48 h post-operative period. Metabolic imaging with FET/FDG scans was utilised for patients with MRI demonstrating multifocal areas of enhancement or a suspicious region of T2Flair/non-enhancing T1 hypodensity. All patients had the diagnosis of WHO 2016 Grade IV glioma confirmed using standard immunohistochemical techniques. O^6^-methylguanine-DNA methyltransferase (MGMT) promoter status was routinely assessed on genomic sequence using pyrosequencing, and potential prognostic molecular profiles using next-generation sequencing for isocitrate dehydrogenase (IDH) mutation, and EGFR amplification was gradually introduced over the study period. Patients with WHO Grade IV Glioblastoma with IDH mutation were not included.

At time of initial assessment by medical and radiation oncology clinic, and then discussion at the MDT, a decision was made whether patient was to be offered adjuvant therapy, and specifically for patients older than 70 years whether they would be considered for management under an elderly protocol. The recommendation to proceed to management under the EORTC-NCIC 60Gy Protocol rather than the Elderly 40Gy Protocol was individualised based upon postoperative factors such as performance status, neurological deficits and extent of residual disease, as well as pre-existing co-morbidities. Patient-informed decision making also was involved in final protocol choice.

Patients were managed under centralised protocols from the Neuro-oncology MDT with dose fractionation schedules per the EORTC-NCIC protocol with 60 Gy being delivered in 30 fractions over a 6-week period [5] or NCIC GBM Elderly protocol with 40Gy in 15 fractions [6]. Treatment technique was IMRT and image-guided radiation therapy in all patients, commencing between day 21 and day 28 post-craniotomy. 

Systemic management followed with TMZ used in two phases: initially at 75 mg/m^2^ daily during the RT, followed by a 4-week break; then 150–200 mg/m^2^ for days 1–5 every 28 days for 6 months [5]. 

### 2.2. Radiological Volumetric Assessment

**Figure 1:**
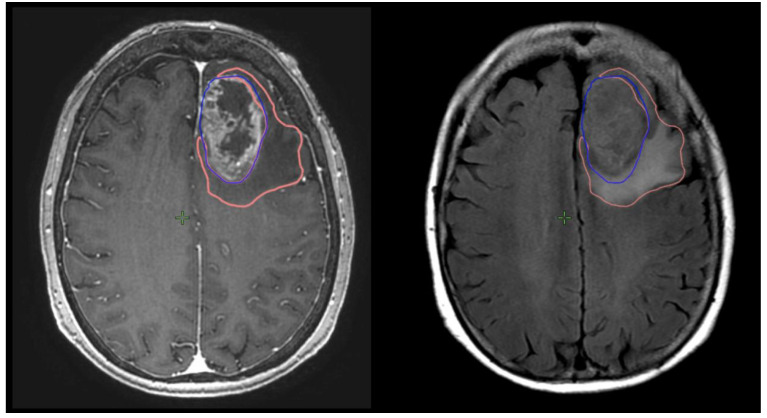
Measurement of T1gd volume (GTVT1gdpre: blue) and T2 volume (GTVT2pre: orange) on respective MRI T1g sequences on right and T2 flair sequence on left.

**Figure 2:**
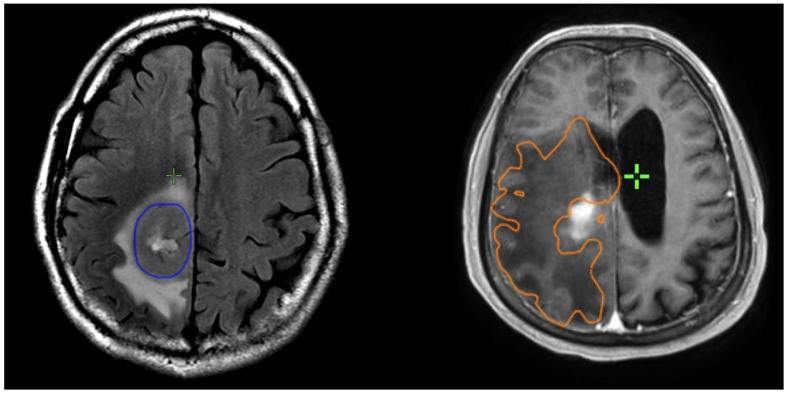
Examples of patients with low T2/T1 ratio on left; and high T2/1 ratio on right. The GTVT1gdpre is shown in blue, and GTVT2pre in orange.

Preoperative and postoperative MRI scans were entered into the radiation planning programme and fused with the registration non-contrast enhanced CT scan. The T1, T2 and T1 gadolinium-enhanced sequences were utilised to manually create volumetric data on both pre- and postoperative scans. The volumes segmented and calculated are demonstrated in Figure 1. These include the preoperative gadolinium-enhancing abnormality (GTVT1gdpre), the preoperative T2 flair abnormality outside of the GTVT1gdpre (GTVT2pre), the postoperative gadolinium-enhancing abnormality subtracted by the blood products on T1 sequence (GTVT1gdpost), and postoperative T2 flair abnormality subtracted by the surgical cavity (T2post). A T2/T1 ratio was calculated from the preoperative volumes (GTVT2pre/GTVT1gdpre), with examples of high and low ratios demonstrated in Figure 2. This T2/T1 ratio was recorded as a continuous variable as well as divided into quartile groups 1–4.

### 2.3. Radiation Therapy Target Volume Delineation

The GTV60 (or GTV40 for Elderly Protocol) was defined as the surgical cavity and GTVT1gdpost. It was expanded to include regions of the T2 Flair (on pre- or postoperative MRI) that were suspicious for macroscopic non-enhancing tumours (defined by increased density of the T2 flair abnormality or uptake on FET PET scan).

The CTV60 (or CTV40) volume was expanded by 5 mm in all directions to anatomical boundaries other than superiorly/inferiorly, where the expansion was 10 mm. The final CTV60 (or CTV40) was expanded by the addition of the postoperative T2 Flair abnormality without any further margin expansion.

The PTV60 (or PTV40) was completed via a 3 mm isotropic margin expansion from the respective CTV60/40.

### 2.4. Follow-Up and Relapse Assessment

All patients were followed closely with initial MRI at 1-month post-RT (M + 1), then second monthly MRI until completion of adjuvant TMZ, three monthly until end of year 3 post-RT, and four to six monthly until progression. Features suggestive of pseudoprogression were actively investigated with sequential MRI or dynamic FET to exclude the risk of true relapse or diagnose the initial site of relapse.

The relapse MRI was entered into the radiation therapy planning system and fused with the original radiation treatment plan. The initial site of relapse was assessed on MRI in relation to site of originally defined GTV60 (or GTV40), generally in relation to the initial surgical cavity and residual T1gad. The relationship of relapse site to initial tumour site is defined as either local (at initial surgical site), distant (separate disease more than 20 mm from initial T1gad abnormality) or combined local and distant. 

Salvage treatments were individualised based on the extent of disease and patient factors but may include repeat craniotomy and second- to third-line systemic therapy.

### 2.5. Statistical Considerations 

The primary endpoint was the relapse-free survival time in months calculated from the time of initial surgical diagnosis of WHO Grade IV glioma to death or date of censure on 1 April 2023. The secondary endpoint was site of progression (either local, distant or combined relapse). Survival curves were generated using Kaplan–Meier method. Univariate predictors of survival duration were evaluated using log-rank comparisons. All reported *p*-values are two-tailed. Statistical significance was defined as *p* < 0.05 in all cases. IBM SPSS Statistics version 23 (IBM Corporation, Armonk, NY, USA) was used for statistical analysis. 

## 3. Results

One hundred and seventy-seven consecutive patients with WHO Grade 4 IDH wildtype glioma, managed with intensity-modulated RT and TMZ between January 2016 and December 2019, were identified from the database and included for analysis. A minimum of 46 months of follow-up was present for all surviving patients.

### 3.1. Baseline Patient and Tumour Characteristics

The median age of patients at diagnosis was 62 years, with 31% of patients aged 70 years or older. Patient, tumour and tumour characteristics are summarised in Table 1. The most common neuroanatomical site of the primary tumour was the temporal lobe (39%), followed by frontal (24%) and parietal lobes (23%). Fifty-nine percent of patients had an ECOG performance status of 0,1 at the start of radiation therapy. 

In particular, a near-total surgical resection was performed in 39% of patients; 47% of patients had tumours that were determined to be MGMT methylated. Three-quarters of the patients were managed under the EORTC-NCIC (Stupp) Protocol with 60Gy (RT60GyDose), whilst the remaining 26% were managed under the NCIC GBM Elderly 40Gy Protocol.

### 3.2. Radiological Volumetric Measurements

The predominant abnormality at diagnosis was an enhancing mass, though 13 patients had predominantly non-enhancing tumours with <1 cm^3^ gadolinium enhancement. The median GTVT1gdpre and T2pre volumes were 20 cm^3^ (q1–3:8–43) and 37cm^3^ (q1–3: 17–70), respectively. For the T2/1 ratio, the median was 2.1, with quartiles defined as Q1 (0–1.0), Q2 (1.1–2.1), Q3 (2.2–5.1) and Q4 (>5.1). 

### 3.3. Overall Survival

One hundred and sixty-two patients have deceased, with a median overall survival of 16.0 months (95%CI:14.1–18.0) and a projected two-year survival of 29.5%. 

Evaluation of potential prognostic factors demonstrated that overall survival was associated with near-total resection (*p* = 0.001); ECOG PS 0,1 (*p* < 0.001); MGMT Methylation status (*p* =0.001) and RT Dose of 60Gy (*p* < 0.001). Age at diagnosis, neuroanatomical site of tumour and Ki67% level were not associated with overall survival. The radiological parameters of initial T1gd volume (*p* = 0.88) and T2 volume (*p* = 0.06) assessed as a continuous variable were not associated with overall survival.

### 3.4. Relapse Free Survival

One hundred and forty-eight patients have relapsed with a median PFS of 11.4 months (95%CI:10.4–12.5). The majority of relapses (74.3%) involved a local component, with 52.7% isolated local relapse. However, the pattern of relapse included a component of distant failure in 43.9%, with 23.6% being an isolated distant relapse. As with overall survival, near-total resection (*p* = 0.02), ECOG PS 0,1 (*p* = 0.007), MGMT Methylation status (*p* < 0.001) and RT Dose of 60Gy (*p* = 0.05) were associated with improved relapse-free survival. As continuous variables, initial T1gd volume (*p* = 0.39) and T2 volume (*p* = 0.23) assessed as continuous variables were not associated with overall survival.

### 3.5. Association of T2/T1 Ratio with Relapse

**Figure 3:**
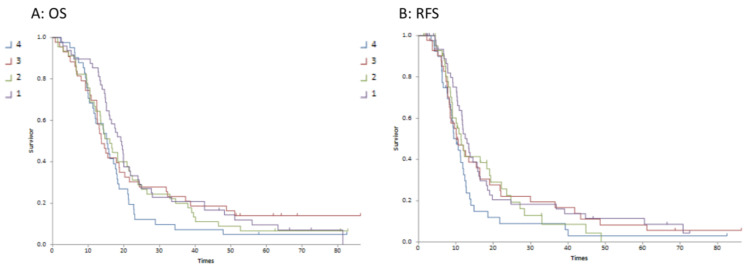
Kaplan–Meier survival curves for T2/T1 ratio quartiles 1–4 (Curve (**A**) is overall survival; Curve (**B**) is relapse-free survival).

Although the continuous variable of tumour volume was not associated with survival outcome, there was a trend to association with T2/T1 quartiles and both OS (*p* = 0.034) and RFS (*p* = 0.024). In particular, the lowest T2/T1 quartile (reflecting a relatively lower T2 volume compared to T1gd volume) was significantly associated with improved overall (*p* = 0.028) and relapse-free survival (*p* = 0.016). Figure 3 demonstrates this association with the difference being between quartile 1 and quartiles 2–4. The median survivals for each quartile are detailed in Table 2.

### 3.6. Association of Radiological Parameters with Distant Relapse

Of the 148 patients with relapse, there were 65 who had a component of distant relapse as part of the failure pattern. There was a trend that the presence of a lower T2/T1 ratio was associated with a lower risk of distant relapse, with hazard ratios for quartiles 1–4 recorded at 0.76, 0.88, 1.03 and 1.56, respectively (*p* = 0.16).

Whilst there was no association of distant relapse with T1gd or T2 volumes as continuous variables, the highest T2/T1 quartile was associated with more distant relapse (*p* = 0.031) compared with the lowest T2/T1 quartile. There was no association of T1gd volume quartiles on distant relapse (*p* = 0.56).

## 4. Discussion

This study demonstrates the varying distribution of T1 and T2 volumes at initial diagnosis between patients diagnosed with glioblastoma, as well as the variation in relative volume within individual patients. Intuitively, the volume of the contrast-enhancing tumour and the amount of oedema relative to the enhancing mass may impact a disease’s natural history. This study demonstrates that the ratio of T2 volume versus T1gad volume is associated with progression-free survival and an increase in the relative amount of distant relapses. In this cohort of consecutively managed patients, the greater amount of T2 flair volume was associated with a higher rate of distant relapse and poor outcome, both in terms of OS and RFS. This may reflect more aggressive pathology producing a greater oedema response or more infiltrative disease along white matter pathways at initial diagnosis. It is important to recognise that the T2 flair volume measured was not overtly suspicious for non-enhancing tumours but rather is a volume generally reflecting oedema.

Although this T2 volume is generally not amenable to aggressive resection either by standard or supratotal resection, the implications may be more important for RT protocol development. Understanding this association with T2 volume theoretically may impact target volume delineation and dose schedules or radiation therapy. Current RT protocols utilise isotropic margin expansion generally around the residual gadolinium-enhancing tumour or surgical cavity without routine coverage of T2 flair abnormality. These protocols are altering with increased uptake of smaller margin expansion as neuroradiological procedures improve the ability to determine non-enhancing disease. However, most protocols remain as isotropic margin expansions and uniform policies not based on neuroanatomical site or appearance.

If a tumour has a relatively higher T1gad component (reflected by a low T2/T1 quartile), then these data suggest potentially the risks of local failure are more significant and attempts to RT dose escalate the known disease of bulk enhancing tumour could be a therapeutic approach. Alternatively, those tumours with more infiltrative patterns suggested by a large T2 component may be at greater risk of distant relapse and, thus, a wider RT volume coverage along white matter tracts or intensification of systemic therapy.

Compared with molecular or genetic studies, basic MRI characteristics such as volumes of enhancing tumour and T2 flair abnormality are an available real-time and reproducible method of predicting a patient’s prognosis. This retrospective study performed in a cohort of uniformly managed patients has demonstrated these features may provide some information regarding patient outcomes, including the site of relapse. More sophisticated MR features, such as relative cerebral blood volume, apparent diffusion coefficient and volume transfer constant may provide prognostic information but become less reproducible or require subsequent processing [7]. Radiomic studies exploring multiple MRI characteristics have explored this concept further as a means of assessing predictive and prognostic factors but remain relatively unavailable [8].

A few retrospective studies have also examined the prognostic value of pre-treatment GBM anatomic volumes [9,10,11,12]. Parameters assessed in these volumetric assessments include tumour volume, necrotic area and oedema area, and images were reconstructed as rectangular, spheroid or ellipsoid [9,10,11]. Iliadis [12] observed that an increase in abnormal T1 volumes is a significant prognostic factor of survival of GBM and found that the preoperative T2 volume has no prognostic significance for survival rates. It should be noted, however, that the sample in this study was from a single institution and was rather small for a multivariate study. 

Saraswathy [13] found that T2 hyperintense volume is significant in the prognosis of GBM patients. This study examined 68 patients and noted that the larger median volume of T2 hyperintense regions was associated with lower survival rates. Zhang [14] also affirmed the statistically significant relationship between the T2/T1 ratio and poorer prognosis. These authors compared the median progression-free survival and overall survival of patients with a dichotomous T2/T1 ratio greater or less than 7.0. Of the 147 patients studied, 63% of those with a low ratio experienced a recurrence with a median PFS of 10 months, and 86.5% of those with a high ratio experienced a recurrence with a median PFS of 6 months. The third of patients with a low ratio had a median OS of 18 months, compared to a median OS of 12 months with a higher ratio. As a continuous variable, there was an association between a high volume ratio and worse PFS and OS [14].

Understanding the potential impact of T2 Flair abnormalities on outcomes, including the site of relapse, emphasises the need to explore methods of incorporating those findings into patient management. This includes both diagnosis of non-enhancing tumour within T2 abnormalities, as well as intervention, including supramarginal resection, radiation therapy target volume delineation or systemic therapies that impact tumour infiltration. Unlike other tumours, GBM infiltrates the brain tissue and forms a gradient of tissue density rather than forming a solid mass [15]. This makes localising the tumour growth with conventional imaging challenging as the infiltrated brain tissues with lower density may appear as normal tissues. To overcome this challenge, additional isotropic margins surrounding the enhancing mass are usually included in radiotherapy planning. These margins typically measure 1.5–2.5 cm [4,16]. However, they are not individualised to each patient, which predisposes patients to radiotherapy-related toxicity and complications such as neurocognitive defects and radiation necrosis [7,17]. This has prompted researchers to investigate other novel imaging techniques that can better identify the invasive extent of the tumours. 

Diffusion tensor imaging (DTI) is an advanced MR technique used to detect the orientation of white matter based on the movement and diffusivity of the water molecules within tissues [18]. Oedema develops around the tumour, and the damage to the blood–brain barrier (BBB) can change the diffusion behaviour of the water molecules in those areas. Furthermore, GBM displaces, disorients and disrupts the white matter fibre, and DTI can delineate the infiltrative GBM along the white matter tracts better than conventional MRI. Since GBM infiltrates preferentially along the white matter tract, the abnormality induced by the tumour can be used to identify occult invasion through DTI [19]. 

MR spectroscopy analyses the change in the resonant frequency of the protons in several brain metabolites using spectral analysis techniques [20]. The metabolites used to characterise brain tumours include N-acetyl aspartate (NAA), choline (Cho), lactates, creatine and lipids [21]. This technique can provide information on tumour growth and necrosis, neuronal activity and cellular membrane synthesis [22]. In gliomas, an elevated level of Cho is observed due to increased cell membrane synthesis, while the level of NAA and creatine is reduced. The concentration values of these metabolites can also be used to differentiate gliomas from metastases, infarcts and abscesses. 

Perfusion-weighted imaging (PWI) is another advanced MR modality; it measures tissue perfusion and vascular permeability [23]. Since the vasculature within tumours such as GBMs is often irregular, PWI can be used to quantify the abnormal vascular environment and yield information regarding tumour grade and treatment response [24]. The dynamic susceptibility contrast technique is the most common technique used in PWI. It examines the decrease in signal following gadolinium-based contrast injection on T2-weighted images [22]. Biological parameters such as relative cerebral blood volume are generated to quantify the amount of blood and, thus, the number of vessels within the tissue. Conversely, dynamic contrast-enhanced (DCE) perfusion can be used to measure the changes in the signal intensity after the contrast injection during T1 relaxation [7]. This technique generates several biological parameters, such as the volumetric transfer constant and fractional volume of the intra- and extracellular space, to estimate the extent and severity of vascular leakage. Another PWI technique is arterial spin labelling, which magnetically labels the blood flowing through the lesion and causes a reduction of signal intensity [22]. 

Positron emission tomography (PET) allows clinicians to gain information regarding the pathophysiology and metabolism of the tumour by intravenously injecting radiotracers that target different metabolic pathways, such as glucose consumption and amino acid uptake. Radiolabelled amino acids such as 18F-fluoroethyltyrosine (FET) and methyl-11C-L-methionine (MET) are commonly used in patients with brain tumours because they have a high tumour-to-background uptake [7]. There is an increased expression of these transporters on the cellular membranes in gliomas, leading to a higher uptake and retention of the tracers intracellularly. The amino acid tracers can pass the BBB independent of its integrity, which allows for the evaluation of non-enhancing tumours with an intact BBB. 

The importance of basic and sophisticated radiological parameters on the treatment design and outcome of glioblastoma is obviously evident. However, integration into future treatment design protocols remains minimal. Understanding basic volume parameters may provide useful information for selecting patients for individualised surgical and radiation therapy protocols.

## 5. Conclusions

This study auditing the radiological volume parameters obtained from basic MRI sequences, specifically, the ratio of the initial T2 volume abnormality to the T1gad volume, provides information on treatment prognosis and pattern of relapse in glioblastoma. 

## Figures and Tables

**Table 1:** Baseline patient, tumour and treatment characteristics.

	Subgroup	Number (n = 177)	Percentage
Age at Diagnosis	<50 yrs	30	17
	>50–69 yrs	92	52
	70–85 yrs	55	31
	Median	62 years	
Tumour Site	Temporal	69	39
	Frontal	43	24
	Parietal	41	23
	Occipital	16	9
	Thalamic	6	3
	Other	2	1
Extent of Resection	Near-Total	69	39
	Subtotal	89	50
	Biopsy	19	11
Ki67%	<20	21	12
	20–29	43	24
	30–49	68	38
	>50	38	21
	Unknown	7	4
MGMT Methylation	No	74	42
	Yes	84	47
	Unknown	19	11
ECOG PreRT	0	38	21
	1	67	38
	2	48	27
	3 or 4	24	14

**Table 2:** T2/T1 ratio quartiles and median overall and relapse-free survivals.

T2/T1 Quartile	Overall Survival	Relapse-Free Survival
1: Ratio <1.0	19.1 months (95%CI: 16.5–21.8)	13.0 months (95%CI: 11.1–14.9)
2: Ratio 1.1–2.1	16.0 months (95%CI: 12.5–19.5)	11.4 months (95%CI: 9.1–13.6)
3: Ratio 2.2–5.1	13.7 months (95%CI: 11.2–16.3)	10.6 months (95%CI: 7.0–14.2)
4: Ratio >5.1	15.8 months (95%CI: 13.2–18.4)	10.6 months (95%CI: 8.2–12.9)

## Data Availability

Data not available due to limitation from Institutional Ethics Review Board.
